# Effects of Second Language Learning on the Plastic Aging Brain: Functional Connectivity, Cognitive Decline, and Reorganization

**DOI:** 10.3389/fnins.2019.00423

**Published:** 2019-05-15

**Authors:** Giovanna Bubbico, Piero Chiacchiaretta, Matteo Parenti, Marcin di Marco, Valentina Panara, Gianna Sepede, Antonio Ferretti, Mauro Gianni Perrucci

**Affiliations:** ^1^Department of Neuroscience, Imaging and Clinical Sciences, “G. d’Annunzio” University of Chieti-Pescara, Chieti, Italy; ^2^Institute for Advanced Biomedical Technologies, “G. d’Annunzio” University of Chieti-Pescara, Chieti, Italy; ^3^Department of Medicine and Science of Aging, “G. d’Annunzio” University of Chieti-Pescara, Chieti, Italy; ^4^Section of Diagnostic Imaging and Therapy, Radiology Division, Department of Neuroscience and Imaging, “SS Annunziata” Hospital, “G. D’Annunzio” University, Chieti, Italy; ^5^Department of Basic Medical Sciences, Neurosciences and Sense Organs, University “A. Moro” Bari, Chieti, Italy; ^6^National Health Trust, Department of Mental Health, Chieti, Italy

**Keywords:** aging, brain plasticity, second language learning, cognitive decline, resting state, functional connectivity

## Abstract

Learning a new language requires the use of extensive neural networks and can represent a powerful tool to reorganize brain neuroplasticity. In this study, we analyze how a 4 months long second language learning program (16, 2 h sessions) can lead to functional changes in the brain of healthy elderly individuals. A large number of studies point out a decline of brain-skills with age; here it is analyzed how cognition together with functional brain organization can be improved later in life. Twenty-six older adults (59–79 years old) were enrolled in the present study. A complete neuropsychological examination was administered before and after the intervention to measure global cognition levels, short- and long-term memory, attention, language access and executive functions. At the end of the program, in the intervention group, the results showed a significant improvement in global cognition together with an increased functional connectivity in the right inferior frontal gyrus (rIFG), right superior frontal gyrus (rSFG) and left superior parietal lobule (lSPL). These findings can be added to the current neurobiological breakthroughs of reshaping brain networks with a short language learning practice in healthy elderly subjects. Therefore, learning a foreign-language may represent a potentially helpful cognitive intervention for promoting healthy aging.

## Introduction

Economic and social implications of pathological aging are dramatically growing (Winblad et al., 2016). Aging is the major risk factor for neurodegenerative diseases and dementia (Niccoli and Partridge, 2012; Alzheimer’s Disease International, 2018). In our society, healthy aging is an objective to be achieved in order to prevent dementia in epidemic proportions. Simple and affordable solutions should be investigated for reducing risks linked to aging, both for the well-being of the individual and caregivers (Lwi et al., 2017). In the course of lifetime, lifestyle factors, such as education, hypertension, diet and depression represent modifiable variables which dramatically impact the risk of pathological aging (Granzotto and Zatta, 2011, 2014; Isopi et al., 2015; Martin Prince et al., 2015; Davies, 2017; Frankish and Horton, 2017; Frisoni et al., 2017; Prince, 2017). It has been shown that older adults can benefit from cognitive and physical interventions (Greenwood and Parasuraman, 2003; Coffman et al., 2014; Ferrucci and Priori, 2014; Luber and Lisanby, 2014; Strenziok et al., 2014). Cognitive and aerobic trainings emerged as potent modulators of cognitive decline (Kivipelto et al., 2013; Ngandu et al., 2015; Firth et al., 2018). Clinical aspects can be supervised and treated with lifestyle factors such as physical exercise or cognitive stimulation. Social stimulation and nutritional components, together with the learning of new cognitive tasks, even late in life, can make the person more autonomous in daily routines and less dependent on caregivers (Granzotto and Zatta, 2011, 2014; Hughes et al., 2012; Isopi et al., 2015; Tan et al., 2016).

Dementia has a multifactorial etiology (Iqbal and Grundke-Iqbal, 2010; Alkadhi and Eriksen, 2011). Recent studies showed how cognitive and brain reserve can prevent detrimental brain aging. Cognitive activities in lifetime boost brain resilience against aging and neurodegenerative disease, this process is known as cognitive reserve (CR) (Schweizer et al., 2012).

In addition, evidence indicates that early bilingualism has defensive effects on our aging brain (Iqbal and Grundke-Iqbal, 2010; Alkadhi and Eriksen, 2011; Boissy et al., 2011; Hughes et al., 2012; Schweizer et al., 2012; Abutalebi and Weekes, 2014; Isopi et al., 2015; Perani and Abutalebi, 2015; Bialystok et al., 2016; Perani et al., 2017). However, it is still debated whether language learning in older monolingual individuals can bring neuroplastic changes on the brain, since life-long bilingual older adults show increased functional connectivity compared to monolingual individuals (Grundy et al., 2017), the investigation focused on the reorganization of distributed brain networks after learning a second language. Few studies have examined differences in language experiences in older adults, especially of a language learning experience later in life (Grady et al., 2015).

Recent works of Grundy et al. (2017), Anderson et al. (2018), DeLuca et al., 2019, and Rosselli et al. (2019) analyzed brain and cognitive modifications effects of bilingualism in young and old adults.

Learning a foreign language could improve cognitive plasticity as this learning task requires the recruitment of extensive neural networks and stimulates different cognitive abilities such as working memory, inductive reasoning, sound discrimination, speech segmentation, task switching, rule learning, and semantic memory (Ware et al., 2017).

Therefore, tests showed that learning a second language during adulthood may exert neuroprotective effects, promote strengthening of brain networks, and improve cognitive reserve (Stern, 2001). Since pharmacological tools with a long-term efficacy to prevent or delay dementia are still missing (Extance, 2010; Collis and Waterfield, 2015; Selkoe and Hardy, 2016), simple and affordable non-pharmacological solutions should be improved in order to train our brain before neurodegenerative condition.

Many studies have investigated the neuroprotective effects of bilingualism in different settings (Gold et al., 2013; Green and Abutalebi, 2013; Gold, 2015; Houtzager et al., 2017), however, it is still largely unknown whether a late intervention is, similarly, effective in monolingual elderly healthy individuals. In addition, functional and structural changes occurring in the brain and underpinning the protective effect of bilingualism have been only partially investigated.

To fill the gap on the effects of late second language learning on brain connectivity, tests concerned the effect of a 4 months intervention focused on learning a second language.

The stimulation of language skills can indirectly stimulate different cognitive abilities (Kowoll et al., 2016; Schroeder and Marian, 2017) and indirectly, eventually counteract detrimental brain aging boosting cognitive abilities. A controlled intervention study, in which 14 healthy Italian-speaking adults were subjected to a 4 months English course, was performed. The objectives of the study were the effects on cognitive status, which were assessed with a comprehensive neuropsychological battery and brain functional connectivity, which was measured by resting-state functional magnetic resonance imaging (rs-fMRI). Subjects underwent rs-fMRI and neuropsychological assessment before and after the language course; the results were compared with those of a control group of monolingual Italian-speaking elderly subjects who did not change their daily habits during the period of the study.

## Materials and Methods

### Study Description

The Ethic Committee of University “G. d’Annunzio” of Chieti approved all procedures and all experiments were performed in accordance with the relevant guidelines and regulations. Thirty participants were recruited from the local community and randomly assigned to one of two groups (1:1) after giving informed written consent. One group was involved in a second language learning course training program, in this case an English course for beginners, which lasted 16 weeks with 120 min of training per week. In the current intervention, each training week consisted of a 1 h and a half classroom session, interspersed with 15 min break and half an hour of homework exercises. The intervention consisted of group lessons with a native teacher. Throughout the intervention, the participants worked on improving their English skills. They acquired basic vocabulary and grammar skills, so they could start communicating in English in everyday social situations. They also learned about British and American English traditions, customs and culture. Participants further developed their speaking and writing skills. They worked on team projects, which provided ample opportunities to practice oral and written communication in English. Participants focused on developing their grammar and vocabulary in areas such as: traveling, shopping and family. All participants were assessed qualitatively by the native teacher at the beginning and at the end of the course.

The control group also completed pre- and post-tests but did not engage in training. In addition, all participants completed a neuropsychological battery prior to and following the training period. Control participants received each month a telephone call to make sure they did not change their lifestyle over the 4 months of the study. According to prior cognitive training research, the battery of tasks measured multiple cognitive abilities, including measures of executive functions, working memory, episodic memory and fluid intelligence. In addition to neuropsychological examination, participants underwent an rs-fMRI acquisition pre- and post-training; the same procedure was applied for the control group.

### Participants

Among the enrolled 30 participants, 26 finished the study (12 in the control group and 14 in the intervention group). Two did not accept to be re-tested at the post-training condition phase, one did not attend enough lessons and another one did not meet inclusion criteria for a re-test. The enrolled participants were right-handed subjects of both genders, aged between 59 and 79 years old. Exclusion criteria were a suspected of cognitive decline after the neuropsychological assessment as well as disorders affecting safe engagement in the intervention (i.e., depression, symptomatic cardiovascular disease, severe self-reported loss of vision, hearing, or communicative ability and coincident participation in another intervention trial, together with any contraindication to MRI scanning, including metal implants and claustrophobia). Smokers and drug abuse subjects were excluded. Participants were asked to refrain from caffeine and alcohol for 24 h prior to the fMRI experimental session to control for external confounders. We randomly assigned participants into the two groups (1:1). Mastery of English proficiency was determined by the teacher during the first meeting through informal conversation and questions concerning the participants’ previous experience with English. All participants were identified as beginner. It is common that Italian elderly people did not have English language lessons during their education. Table 1 describes participant characteristics.

**Table 1:** Participants demographics.

	Control group	Intervention group	
	(*n* = 12)	(*n* = 14)	ANOVA one-way
Age (mean, *SD*)	65.7 (3.7)	69.5 (5.3)	*F =* 4.42, *p* = 0.04
Education	13 (2.7)	9.6 (2.9)	*F* = 8.43, *p* = 0.008
Sex	5M/7F (*Y*. = 41.7)	2M/12F (*Y*. = 14.3)	*X*^2^ fisher corrected *X*^2^ = 2.46, *p* = 0.19

### Conditions

**FIGURE 1:**
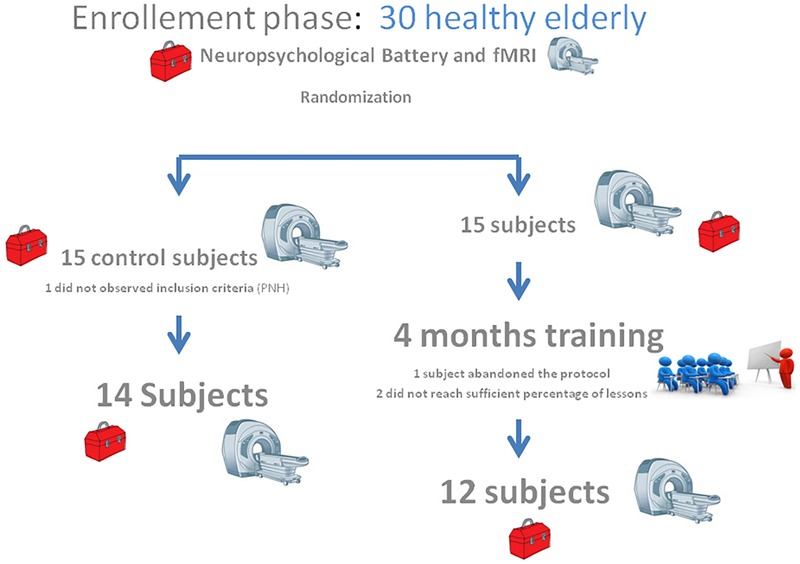
Study design. The pictogram illustrates the study design paradigm.

Participants in the intervention group (12 female, 2 male; average age = 69.5) underwent a learning program located in the Abruzzo region. Classes were held by a native English teacher. Participants in the control group (7 female, 5 male; average age = 65.66) did not change their daily routine during the 4 months. Subjects were called monthly; an informal interview was used to ask for changes in lifestyle. All participants completed the same pre- and post-cognitive evaluation as well as the rs-fMRI acquisition (for study design see Figure 1).

### Behavioral Assessment

A comprehensive neuropsychological assessment to investigate different cognitive abilities was performed at baseline and after the 4 months intervention. All subjects included in the study, of both control and intervention groups, completed the tests for cognitive domains scores. The battery included measures of: global cognition with a Mini Mental State Examination (MMSE) test (Measso et al., 1993); attention, in the present case sustained spatial attention evaluated by Trial Making Test (TMT) A; divided spatial attention evaluated by with TMT B; cognitive flexibility TMT AB (Giovagnoli et al., 1996); phonological lexicon access with a Verbal Fluency Test (FAS), which is also a measure of the executive functioning domain (Novelli et al., 1986; Bianchi and Dai Prà, 2008); short and long term episodic memory with Babcock Memory test (Carlesimo et al., 1996) and executive functions with the Frontal Assessment Battery (FAB) test (Lezak and Lezak, 2004; Appollonio et al., 2005). This procedure was repeated at the post-test phase and these scores were analyzed using Statistical 8 and Statistical Package for Social Sciences (SPSS, Inc, Chicago), version 15.0.T.

### Behavioral Analysis

Arithmetic mean and standard deviation, as well as median, percentage and range were used to report the general characteristics of the study population and controlled between group using *t*-test or Chi-square statistic (Table 1). To compare the intervention group and the control group at enrollment, general linear model statistical test was performed. The analyzed outcomes were the pre- and post-differences in performance. To indicate statistical difference, two-tailed *P*-value of less than 0.05 was considered. The baseline cognition was included as a covariate. The significance threshold was further adjusted for multiple comparisons using Bonferroni’s correction. This data analysis was carried out using the software Statistica 8.

### Imaging Procedure

Images were acquired with a Philips Achieva 3 Tesla scanner (Philips Medical Systems, Best, Netherlands) using a whole-body radiofrequency coil for signal excitation and an 8-channel phased-array head coil for signal reception. A high-resolution structural volume was first acquired using a 3D fast field echo T1-weighted sequence (sagittal, matrix 240 × 240, FOV = 256 mm, slice thickness = 1 mm, no gap, in-plane voxel size = 1 × 1 mm, flip angle = 8°, TR = 8.2 ms and TE = 4 ms). Afterward, the data from Blood Oxygen Level Dependent (BOLD) fMRI were obtained using a gradient-echo T2^∗^-weighted echo-planar (EPI) sequence with the following parameters: matrix 64 × 64, voxel size 3.6 mm × 3.6 mm × 5 mm, SENSE 1.8, TE = 30 ms, TR = 1.1 s. Three runs were acquired, with 300 volumes per run. During fMRI, cardiac (ppu) and respiratory (belt) data were also acquired. Physiological signals were recorded using a pulse oximeter placed on a finger of the left hand and a pneumatic belt strapped around the upper abdomen. Cardiac and respiratory data were both sampled at 100 Hz and stored by the scanner software in a file for each run.

### fMRI Data Pre-processing

AFNI Software was used to perform the analysis of fMRI data^1^. To allow T1 balancing equilibration of the MR signals, the first five volumes of each functional run were discarded. First, despiking (AFNI’s “3d Despike”) was performed to remove transient signal spikes from the EPI time series, followed by RETROICOR (Glover et al., 2000) to remove signal fluctuations related to cardiac and respiratory cycles and slice scan time correction. Motion correction was performed using rigid body registration of EPI images to the sixth volume of the first run. To remove further physiological and hardware related confounds, ANATICOR (Jo et al., 2010) was employed for additional pre-processing. A global nuisance regressor was obtained extracting the EPI average time course within the ventricle mask and local nuisance regressors were obtained calculating for each gray matter voxel the average signal time course for all white matter voxels within a 3 cm radius (Jo et al., 2010). AFNI’s @ANATICOR was used to remove nuisance regressors and the six regressors derived from motion parameters from the EPI timeseries of each run. Structural scans segmentation done by FreeSurfer^2^ permitted to obtain individual masks of large ventricles and white matter. Then we performed a co-registration to EPI using an affine transformation.

Finally, preprocessed functional scans were normalized to the MNI space, spatial smoothing (6 mm FWHM), and band-pass filtering (0.01–0.1 Hz) were performed. The framewise displacement (FD) and the root average square value of the differentiated BOLD timeseries were calculated (DVARS) within a whole brain spatial mask. Quality control measures to inspect between-groups differences of motion effects, which could potentially not be calculated, were added in the special registration and regression of motion parameters accounted by spatial registration and regression of motion parameters (Power et al., 2012, 2014).

### Functional Connectivity Analysis

First of all, seed-based resting state connectivity maps were created for individual subjects calculating the Pearson correlation coefficient (*r*-value) between the Posterior Cingulate Cortex (PCC) of the Default Mode Network (DMN) time series and the time series at each voxel. The PCC time series was derived by averaging the time courses of voxels inside a sphere with a 6 mm radius (Table 2). Individual correlation maps were converted using the z-Fisher transformation (z = atanh (r), where r is the correlation coefficient) to approach a normal distribution before calculating the random effect group analysis.

**Table 2:** Principal brain networks investigated in MNI coordinates.

					Brain
*x*	*y*	*z*	Seed	Brain region	network
0	-51	29	PCC	Posterior cingulate cortex	DMN
-32	16	-8	LAIFO	Anterior insula frontal operculum	SLN
27	3	57	FEF	Frontal eye field	DAN
-56	-44	22	LTPJ	Temporal parietal junction	LAN
1	-87	-2	LG	Lingual gyrus	VIS
-51	-18	7	L STG	Superior temporal gyrus	AUD
-52	-9	31	PCG L	Pre-central gyrus	MOT
-51	-54	37	LIFC	Bilateral parietal cortex	FPCN
-13	-14	4	IPG	Inferior parietal gyrus	CEN

A one-sample *t*-test was performed on the z-Fisher maps to obtain group statistical functional connectivity maps, separately for the control and the intervention groups. These group statistical maps were thresholded at *p* < 0.05 corrected for multiple comparisons using False Discovery Rate (FDR) and were utilized to visually inspect the level of connectivity for the two groups. Then, to quantify statistically significant differences across groups and time, a number of spherical nodes (6 mm of radius) for each region, which is known to have a correlation, were defined using independent coordinates from the literature (see Table 2). The examined nodes have been chosen to be correlated, that is they increase their activity simultaneously, or anti-correlated, i.e., they decrease their activity with the PCC (Esposito et al., 2018).

This procedure was utilized in order to avoid circularity problems in the analysis (Kriegeskorte et al., 2009). Individual connectivity values were extracted from these regions of interest (ROI) and compared across groups and conditions using a repeated measure analysis based on multivariate modeling (MVM) approach as implemented in R Software (Chen et al., 2014). A multivariate, seed-based approach was employed to assess functional connectivity in brain networks simultaneously by including a seed for the DMN (the posterior cingulate cortex, or PCC). This seed-approach is useful for distinguishing network activity between groups of participants, and for distinguishing connectivity patterns that differ across brain regions (Campbell et al., 2013). Although there are conflicting opinions (Mineroff et al., 2018; DeLuca et al., 2019), multifaceted cognitive abilities (and language is one of those) depend on multiple mental processes that engage different large-scale functional networks including parietal, frontal and temporal cortical regions of the brain. In particular, executive processes involved in second language learning (including cognitive control, semantic processing, and working memory) are supported by DMN, executive control network (ECN) and language network (Varela et al., 2001; Woolgar et al., 2017). Since the DMN has been shown to be involved in age related changes, which are reflected in both within and between network connectivity modifications (Wu J.-T. et al., 2011; Esposito et al., 2018), the PCC (which is considered the main hub of the DMN) was chosen as a seed region for our analysis.

In contrast, choosing a seed region of the language network could have limited the investigation of potential plasticity effects due to the present training in regions not strictly linked to linguistic aspects.

Furthermore, semantic/conceptual processing engages regions of both DMN and language network (Chen et al., 2006; Toro et al., 2008; Uddin et al., 2009).

Data were analyzed with a linear mixed effects model in R3, which estimates both parameters using Maximum Likelihood Estimation and effects using specific contrast matrices. The fixed factors were defined as the group (control versus intervention) and time (T0 versus T1), and the subject of either group was entered as a random factor. By considering the nine ROIs, the number of statistical tests which were performed were 18 comparisons. To prevent Type I error, contrasts were both assessed at *p* < 0.05 corrected for Bonferroni multiple comparisons.

### fMRI Data Analysis and Cognition

Pearson’s correlation analysis was performed in each group separately to examine the association between cognition (MMSE corrected score for age and education) and functional variables (connectivity signal variation in regions showing between-group effects). Sex and educational level were also included as a co-variate. Statistical Package for Social Sciences (SPSS, Inc, Chicago), version 15.0.T was used for the purpose. Statistical significance for correlation analysis was set at *p* < 0.05, corrected for multiple comparisons using Bonferroni correction.

## Results

Results of the study indicate that a 4 months second language learning intervention improves global cognitive performances and reorganizes functional connectivity.

### Cognitive Performances

**FIGURE 2:**
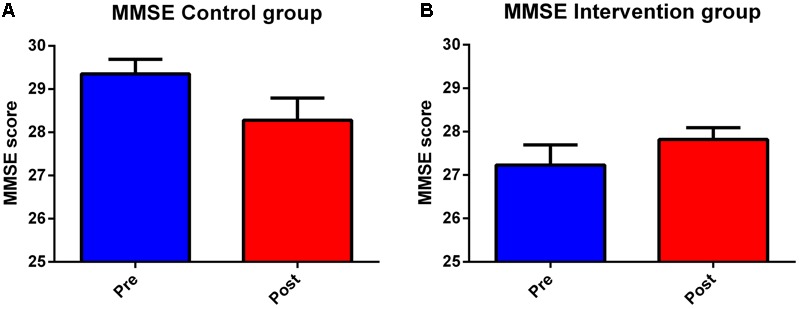
Second language learning positively affects global cognition performances. Histograms depict results of neuropsychological evaluation in control **(A)** and intervention **(B)** groups at the beginning of the study (Pre) and after 4 months (Post). Graphs show results, expressed as means of MMSE and SEM. The trained group shows, after 4 months (T1), a statistically significant improvement compared to control group.

Control and intervention subjects were evaluated at the baseline phase (T0) and at the end of the 4 months (T1) period for their neuropsychological abilities. Four subjects were excluded, one did not observe inclusion criteria [had periventricular nodular heterotopia (PNH)], two did not accept to be retested at post-training condition, and one did not attend a sufficient percentage of lessons. We observed slight differences between group in terms of age (Control group Mean: 65.7, SD 3.7; Intervention group: Mean 69.5, SD 5.3; One-Way ANOVA *F* = 4.42, *p* = 0.046) and education (Controls: Mean 13.0, SD 3.0; Intervention group Mean 9.6 SD 2.9; One-Way ANOVA *F* = 8.43, *p* = 0.0008). A detailed description of statistical analysis results can be seen in Table 1. The normality of the distribution was controlled by Kolmogorov–Smirnov test (Ksd *d* = 0.11, *p* > 0.20). Statistically significant differences in MMSE score were found within and between the two groups at both T0 and T1 (*p* = 0.009). In more details, the two groups significantly differ at T0, with the control group performing better than the intervention group (29.35 versus 27.23, Duncan *post hoc p* = 0.001); on the contrary, the between group difference disappeared at T1 (28.28 versus 27.81, Duncan *post hoc p* = 0.42). In fact, only the control group significantly decreased its performances over time (29.35 versus 28.28 Duncan *post hoc p* = 0.017), whereas the intervention group remained stable (27.23 versus 27.81) (see Table 3 and Figure 2). Since language is a task that involves many cognitive abilities, several cognitive domains were investigated for this purpose, but all these domains were involved in the aging-related cognitive decline. Therefore, the performance in prose memory was then studied using the prose memory test (Babcock story version A) (Horner et al., 2002), a test that investigates short-term and long-term memory. The performance of both groups in their attention skills was evaluated by using TMT (Giovagnoli et al., 1996), a test that analyzes visual attention and task switching. The results of different subsets, A and B (Test-A: sustained attention; Test B: divided attention; and Test B-A: task coordination and set-shifting) were also analyzed.

**Table 3:** MMSE values; Group 0 is referred to Control group while Group 1 is referred to Intervention group.

MMSE score				
	**Group 0**		**Group 1**		
	**Mean**	***SD***	**Mean**	***SD***	

T0	29.35	1.18	27.23	1.72	
T1	28.28	1.76	27.81	1.01	

	***F***	***DF***	***P***	**Duncan *post hoc* effect**	

Within effect (Time)	0.68	1.24	0.419	0.549	–
Between effect (Group)	6.88	1.24	0.015	0.015	Group 0 > Group 1
Quadratic interaction	7.98	1.24	0.009	0.017	Group 0 T0 > Group 0 T1
(Group × Time)
				0.17	Group 1 T0 = Group 1 T1
				0.001	Group 0 T0 > Group 1 T0
				0.422	Group 0 T1 = Group 1 T1

Moreover, Frontal Assessment Battery (FAB) (Dubois et al., 2000; Cyarto et al., 2010) test and Verbal Fluency (FAS) (Tombaugh et al., 1999; Costa et al., 2014) test were performed: they are two tests that are employed to evaluate the functioning of frontal lobes (FAB) as well as attention or lexical production (FAS) (see Table 4).

**Table 4:** Neuropsychological performances.

	Intervention T0	Intervention T1	Controls T0	Controls T1	*p*
	Mean/*SD*	Mean/*SD*	Mean/*SD*	Mean/*SD*	
**Global cognition**		
MMSE	27.23/1.66	27.81/0.97	29.35/1.13	28.28/1.68	0.009^∗^
**Speed attention**		
TMT A	33.78/27.74	26.90/16.40	62.91/42.11	49.91/23.18	0.61
TMT B	58.35/68.16	55.85/60.99	83.25/38.39	72.66/28.80	0.62
TMT AB	18.60/31.69	25.95/54.41	20.83/61.50	29.08/11.67	0.96
**Immediate and delayed verbal memory**		
IR	6.75/1.95	7.67/0.98	6.37/1.50	7.08/1.47	0.79
DR	6.73/1.50	7.64/1.08	6.36/1.43	6.83/1.34	0.55
**Language**		
FAS	36.5/6.86	37.42/8.53	34.42/9.46	35.3/9.13	0.98
**Executive functions**		
FAB	17.10/1.32	17.14/1.47	17.1/1.2	17.04/1.53	0.90

*Means and standard deviation. ^∗^Indicates *p* < 0.05.*

Finally, the subject autonomy for daily and instrumental activities (ADL, IADL) (Song et al., 2014) was investigated. No statistically significant differences were found within and between the groups for these tests (see Supplementary Figure 1).

### Brain Functional Connectivity

**FIGURE 3:**
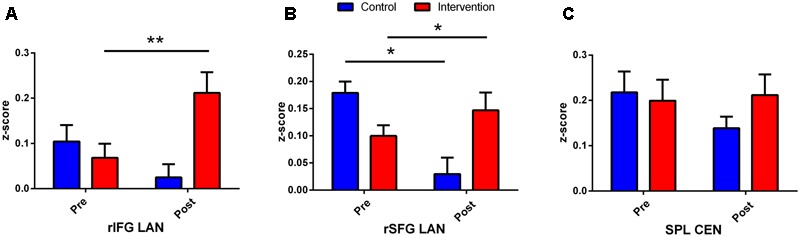
Second language learning improves neuronal connectivity: effects in the Language Network (LAN) and in the Central Executive Network (CEN). The graphics depict the connectivity values from rIFG **(A)**, rSFG **(B)**, and SPL **(C)** extrapolated from control and intervention group difference (T1–T0). ^∗^Indicates *p* < 0.05 and ^∗∗^indicates *p* < 0.01.

**FIGURE 4:**
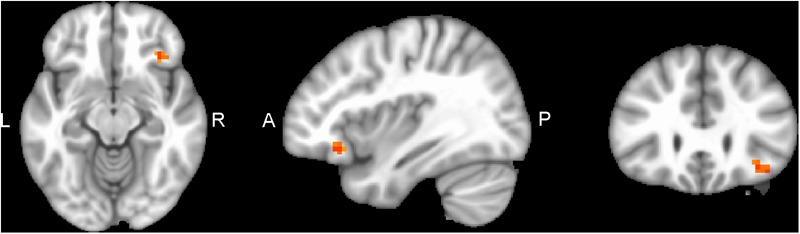
rIFG of LAN network connectivity, non-radiological system L = L. Results from the right inferior frontal gyrus (rIFG, MNI 35.5 27.5 -11.5) from multivariate modeling (MVM) approach correlation analysis is displayed. BOLD connectivity showed a greater increase in the rIFG from pre- to post-training (*p* < 0.05, FDR corrected).

**FIGURE 5:**
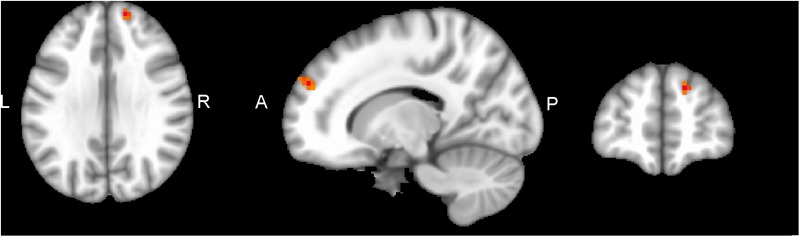
rSFG of LAN network connectivity, non-radiological system L = L. Results from the right superior frontal gyrus (rSFG MNI 14.5 54.5 30.5) from multivariate modeling (MVM) approach correlation analysis is displayed. Connectivity values showed a greater increase in the rSFG from pre- to post-training (*p* < 0.05, FDR corrected).

**FIGURE 6:**
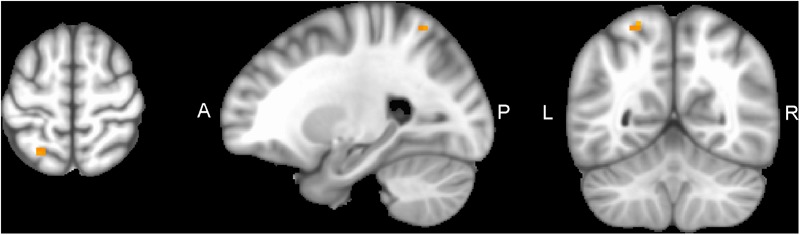
SPL of CEN network connectivity, non-radiological system L = L. Results from the superior parietal lobule (SPL MNI -24.5 -56.5 60.5) from multivariate modeling (MVM) approach correlation analysis are displayed. Connectivity values showed a greater increase in the SPL from pre- to post-training (*p* < 0.05, FDR corrected).

Performing MVM FMRI interaction analysis between baseline (T0) and after 4 months (T1) in both groups (ANCOVA interaction, *p* = 0.001, FDR corrected), significant connectivity changes in specific areas of language network (LAN) and control executive network (CEN) were found (Figure 3). All FMRI data were controlled for age and education, entering these variables as co-variates. Significant (*p* < 0.001) longitudinal increases were found in the intervention group in the LAN for the strength of functional connectivity in the right inferior frontal gyrus (rIFG) (MNI 35.5, 27.5, -11.5) and in the right superior frontal gyrus (rSFG) (MNI 14.5, 54.5, 30.5) regions (*t*-test between T0 and T1, *p* = 0.001, *t* = 3.703, Figure 4, 5). Moreover, CEN network revealed a change in the strength of functional connectivity in the trained group in the superior parietal lobule (SPL), (MNI -24.5 -56.5 60.5), (SPL; *t*-test between T0 and T1 in the training group, *p* = 0.001; *t* = 3.703, Figure 6).

Analysis of the other Resting State Networks (RSNs, Table 2) did not show significant modifications.

Finally, a Pearson Correlation analysis between significant neuropsychological results and fMRI data was performed.

The relationships between longitudinal functional connectivity changes and global cognition changes were assessed by extracting connectivity values from the mean values of the voxels in the cluster showing a group × time interaction effect on connectivity.

For the trained group, a positive significant correlation between the strength of functional connectivity in rSFG and the global cognition abilities (Pearson *r* = 0.4255, *p* = 0.03) was found.

**FIGURE 7:**
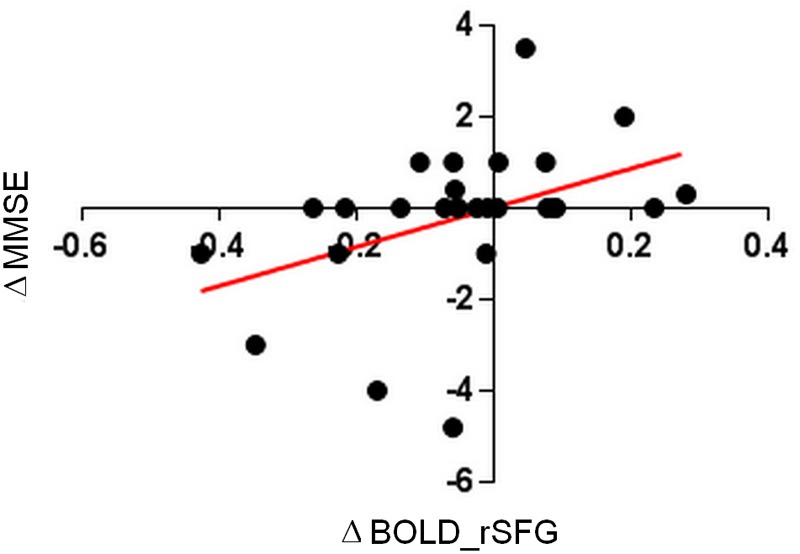
Pearson correlation of global cognition differences between T1 –T0 (ΔMMSE) and connectivity differences values for right superior frontal gyrus (ΔrSFG).

Changes in connectivity values correlated positively with changes in MMSE score, and all this supported the hypothesis that all the observed changes in the treated group were driven by the language learning course exposure and not by the 4 months time interval. A detailed description of correlation analysis is shown in Figure 7.

To consider the possible confounding effect of differences in MMSE between groups at baseline phase, the MMSE at T0 was entered as a co-variate in the general linear model analysis. Also in this case, results remained significant in the rIFG [*p* = 0.02, *F*(1, 23) = 5.68] and in the rSFG [*p* = 0.04, *F*(1, 23) = 4.70] instead SPL showed only a statistical trend [*p* = 0.08, *F*(1, 23) = 3.13].

Moreover, to verify if the MMSE differences were linked to connectivity changes, GLM between Δ (T1 minus T0) connectivity values for LAN and CEN clusters and the Δ MMSE was performed.

Results were significant for rSFG [*p* = 0.012, *F*(1, 23) = 7.35] and for rIFG [*p* = 0.013, *F*(1, 23) = 7.08]; SPL showed, however, a trend [*p* = 0.08, *F*(1, 23) = 3.13] Yates corrected Chi-square. (Figure 3)

Only in the intervention group there was a significant change in both functional and behavioral measures after intervention, thus supporting the initial hypothesis that language learning training can improve cognition in healthy elderly subjects.

The present data were also controlled for age and educational level, in this case results were significant for rIFG [*p* = 0.024, *F*(1, 21) = 5.89], instead rSFG showed, however, a trend [*p* = 0.07, *F*(1, 21) = 3.59] and SPL was not significant for this condition [*p* = 0.39, *F*(1, 21) = 0.73].

## Discussion

The present study aimed at investigating neuroplastic-related effects of second language learning in terms of cognitive and brain networks functional connectivity changes. For this purpose, two groups of healthy elderly undergoing 4 months of second language learning course were tested. Resting-state fMRI was employed to observe effects on brain functional connectivity and cognition. Short-term longitudinal changes in functional connectivity together with an improvement in global cognition were also observed. Three new findings can be detected through the present study: an increase in global cognition scores in the intervention group (1), a significant short-term increase in functional connectivity in language network and CEN. The present work showed a significant longitudinal increase in the right inferior frontal gyrus and in the rSFG regions. Analysis of the CEN revealed a change in the strength of functional connectivity in the superior parietal lobule (2). Rate of change in global cognition was positively correlated with functional connectivity improvements, suggesting a relationship between behavioral change and functional alterations (3). There are several studies showing an improvement in cognition after physical or cognitive training or combined (Colcombe et al., 2006; Voss et al., 2010; Erickson et al., 2011; Kivipelto et al., 2013; Carlson et al., 2015; Ngandu et al., 2015). As far as one can know, this is the first study demonstrating an impact of short-term second language learning on cognition along with functional connectivity of language and control network in aging. Studies report brain connectivity changes in response to cognitive learning, motor training or both (Pieramico et al., 2012; Antonenko and Flöel, 2014). Nevertheless, as far as the authors’ know, functional neuroplastic effects of a short language learning intervention in healthy elderly individuals, have not been analyzed with functional magnetic resonance (fMRI) methods (Antoniou et al., 2013).

The present results showed increased functional connectivity in the language network, in particular in right inferior frontal gyrus and rSFG, together with the left parietal lobule of the control network. These regions are critical for different processes. The right inferior frontal gyrus, which is involved in inhibition and attentional control, also known as Brodmann Area 44, has been implicated in go/no go tasks (Hampshire et al., 2010), more specifically it runs impulse control through inhibition. This kind of process is needed in the switching between two languages in bilingual people, and it has been shown that it can protect brain in dementia condition (Luk et al., 2011b; Green and Abutalebi, 2013; Costa and Sebastián-Gallés, 2014; Rossi and Diaz, 2016). The rSFG is involved in control of impulsive response, a hallmark of cognitive control (Hu et al., 2016). The superior frontal gyrus (SFG) is located at the superior part of the prefrontal cortex and is involved in a variety of functions, it has also been parcellated in subregions which are: anteromedial (SFGam), dorsolateral (SFGdl), and posterior (SFGp) subregions which were divided according to the diffusion tensor tractography (Li et al., 2013; Hsiao et al., 2015). The SFGam is anatomically connected with the anterior and mid-cingulate cortices, which are critical nodes of the cognitive control network and the default mode network. The SFGdl was connected with the middle and inferior frontal gyri, which are involved in the executive network. The SFGp was connected with the precentral gyrus, caudate, thalamus, and frontal operculum, which are nodes of the motor control network. Resting-state functional connectivity analysis further revealed that the SFGam is mainly correlated with the cognitive control network and the DMN; the SFGdl was correlated with the cognitive execution network and the DMN; and the SFGp was correlated with the sensorimotor-related brain regions. The LPL is involved in retrieval of learnt facts and these are also involved in conceptual decisions on object names (Cappelletti et al., 2010). The present results are in line with and corroborate previous studies indicating the role of those brain regions in control processes. Also in bilingual or multilingual people, between-language competition requires neuronal effort to suppress activation of the non-target language (Marian et al., 2014, 2017). This neural effort is supposed to be involved in protecting against pathological aging and in the dementia delay (Abutalebi and Weekes, 2014; Alladi et al., 2014; Bak et al., 2014; Bialystok et al., 2016).

It has already been discussed that functional brain connectivity may be sensitive to disease-specific network changes in neurodegenerative diseases (Pievani et al., 2011). Assessed with resting-state connectivity, fMRI has shown distinct patterns of network disruption across the major neurodegenerative diseases.

Different works show how default mode network undergoes functional anomalies in Alzheimer Disease (AD) (Wu X. et al., 2011) or functional changes in the salience network in frontotemporal dementia (Filippi et al., 2017).

Properties of brain networks in healthy controls, compared to patients with behavioral variant of frontotemporal dementia (bvFTD) and patients with early-onset Alzheimer disease (EOAD) has been observed with graph analysis, a method for the analysis and representation of complex networks. In those studies, DMN is crucially impaired in AD, whereas ECN, dorsolateral prefrontal attention network, and semantic appraisal network are impaired in bvFTD. It can be hypothesized that the observed changes in LAN and CON in healthy aging can indirectly modify the functional connectivity of DMN together with other main brain networks. The brain is intrinsically organized into dynamic, correlated and anticorrelated functional networks, second language learning can longitudinally preserve principal network from deterioration (Fox et al., 2005). Moreover, plastic changes in the language network are, in the present work, lateralized to right hemisphere. Language functions are normally lateralized to the left hemisphere for right handed individuals. However, plasticity changes in the right hemisphere were observed. This could be expected in the case of second language acquisition whose process showed in adults right hemisphere involvement as well (Briganti et al., 2012; Plante et al., 2015). Moreover, other studies on bilingualism have provided evidence of reduced left lateralization (i.e., greater right hemisphere participation) for verbal tasks performed using the second language rather than the first, suggesting that the right hemisphere plays a role in the early stages of both child and adult language acquisition (Oxford and Ehrman, 1995).

This result can be explained with recent findings indicating that learning a second language triggers the recruitment of contralateral brain areas. With diffusion tensor imaging (DTI) measuring resting-state functional connectivity in monolingual and bilingual older adults, Luk et al. (2011a,b), showed higher white matter integrity in bilingual older adults, primarily in the corpus callosum connecting the two hemispheres but also extending to bilateral superior longitudinal fasciculi, right inferior frontal-occipital fasciculus and uncinate fasciculus. Luk and colleagues, with a resting-state functional connectivity analysis, showed that, while both monolinguals and bilinguals had correlating brain activity with contralateral regions at rest, bilinguals had increased anterior-posterior connectivity.

The present work shows that the approach of characterizing the brain as a network using rs-fMRI and MVM analysis can provide new insights into how language learning affects brain function and functional connectivity in aging.

Several protocols have been used with the purpose of maintaining healthy cognitive functions (Cotlarciuc et al., 2016; Brem and Sensi, 2018). In a recent study, Ware et al. (Ware et al., 2017) have shown that computer-assisted learning of a second language leads to social and motivational benefits, although they did not investigate different cognitive domains. In the present study, subjects were tested with a comprehensive battery for cognitive abilities as well as for changes in brain functional connectivity. Brain plasticity and potential reorganization against behavioral and functional brain deterioration were investigated. These results are in line with studies showing changes in brain reserve and metabolic connectivity in bilinguals (Li et al., 2017; Perani et al., 2017). The results of the analysis indicate beneficial effects of second language learning late in life on global cognition. Despite starting from a lower global cognitive level, the intervention group reached a higher global cognition improvement (MMSE T0 MEAN = 27.23, *SD* = 1.66; T1 MEAN = 27.81, *SD* = 0.97), in comparison to the control group (T0 MEAN = 29.35, *SD* = 1.13; T1 MEAN = 28.28, *SD* = 1.68).

Learning a new language, also late in life, is a big cognitive challenge (Blumenfeld et al., 2017). The present work supports the idea that the aging brain is a dynamic set of biological features that can plastically reorganize against pathological decline. Achieving positive results is possible thanks to a reorganization of a set of brain mechanisms, including adult neurogenesis, synaptic changes, dendritic branching, axon sprouting or changes in the number and morphology of glia cells, for both astrocytes and microglia (Wenger et al., 2017).

In the field of non-pharmacological stimulation against pathological aging, the present results suggest that rs-fMRI can be used to detect connectivity changes after a period of 4 months, demonstrating sensitivity of BOLD signal as an imaging biomarker for functional connectivity short-term changes in aging. Thanks to the adaptive and plastic structure of our brain, even late in the elderly, the brain is able to respond dynamically to cognitive challenges. It remains to be investigated whether such brain changes will be maintained over time.

However, some technical limitation must be included. The size of the present cohort is relatively small, and the training effects of the intervention group were compared with those of a passive, but not an active, control group.

Collectively, our findings show that just 4 months of learning a second language leads to functional reorganization processes in the mature human brain together with an improvement in global cognition. These findings crucially complement current neural concepts of neuroplasticity in aging brain, a condition that can delay any pathological cognitive process and dementia. The current study gives a contribution in the field of brain training. The concept of brain and cognitive reserve, that is the brain resilience capacity (Stern, 2012), become a resource that could be one shock-absorber for pathological aging and can be increased even later in life.

The present study confirms the ability-capability of the aging brain in reorganizing neural networks maintaining and even improve mental functioning despite aging.

## Conclusion

In conclusion, the present results demonstrate that longitudinal changes in functional connectivity and in cognition can be detected over an interval of merely 4 months in middle-aged and older adults. Furthermore, connectivity changes differed between the control and the intervention groups, suggesting a positive impact of second language learning on short-term functional connectivity trajectories.

Change in global cognition performances addressed by the present intervention, correlated positively with the rate of change in the bold signal. This can support rs-fMRI as a behaviorally relevant imaging biomarker.

Aging is paralleled by an increase in deterioration of cognitive abilities, however, with a simple, short and economic training, robust effects in terms of brain connectivity, global cognition functioning, and brain plasticity can be provided. Challenging aging with a learning stimulation can be a powerful tool to reorganize neuronal networks and cognitive behavior with the involvement of boosted neuronal activity.

Brain dynamism of the aging system can be more consistent and can bring global improvement taking advantage of mechanisms like cognitive reserve and functional plasticity (Greenwood and Parasuraman, 2003).

A simple and short cognitive intervention can be designed to improve cognition supported by the reorganization of functional brain circuitry and the increase in neuronal structures. A picture of a static decline with aging can be easily improved with a dynamic way of life, by means of stimulation to continuous learning of new knowledge and with healthy and dynamic lifestyles. These results should consider that a second language learning program, even late in life, can be considered a non-pharmacological treatment able to counteract cognitive aging along with the onset of dementia. Learning a second language is a powerful tool that can be part of a healthy lifestyle program that can preserve brain plasticity in aging individuals. Further studies are needed to explore whether these improvements are long-lasting or are reverted at the end of the training period.

## Ethics Statement

This study was performed in accordance with the recommendations of the Declaration of Helsinki. All subjects signed written informed consent. The protocol was approved by the Ethics Committee of G. d’Annunzio University of Chieti, Italy.

## Author Contributions

GB conceived the idea, designed the research and wrote the manuscript. GB, MdM, MP, AF, and MGP supervised the experiments, exported the data, and reviewed the manuscript. PC and GS ran the statistical analysis. MP and MGP supervised the research and reviewed the manuscript for intellectual content. VP did the neuroradiological examinations. All authors approved the final version.

## Conflict of Interest Statement

The authors declare that the research was conducted in the absence of any commercial or financial relationships that could be construed as a potential conflict of interest.

## Footnotes

1 afni.nimh.nih.gov/afni

2 http://surfer.nmr.mgh.harvard.ed

## Supplementary Material

FIGURE S1 Second language learning affects cognitive performances. Histograms depict measures of neuropsychological evaluation in control and intervention groups at the beginning of the study (Pre) and after 4 months (Post). Graphs show results, expressed as means and SEM for the performance of both groups in their sustained attention skills using TMT_A test for controls **(A)** and interventions **(B)**, divided attention (TMT_B) for controls **(C)**, and interventions **(D)**; task coordination and set-shifting (TMT_BA) for controls **(E)** and interventions **(F)**. Global prose memory test (Babcock story, version A) was also performed, with immediate recall (IR) **(I,J)** and delayed recall (DR) subtest **(M,N)**. Scores for functioning of frontal lobes (FAB) are represented for controls **(K)** and intervention group **(L)**. Differences for scores for lexical production (FAS) are also displayed **(G,H)**, together with ADL **(O,P)** and IADL scales **(Q,R)**.
