# Corrigendum: Effects of Second Language Learning on the Plastic Aging Brain: Functional Connectivity, Cognitive Decline, and Reorganization

**DOI:** 10.3389/fnins.2020.00108

**Published:** 2020-02-19

**Authors:** Giovanna Bubbico, Piero Chiacchiaretta, Matteo Parenti, Marcin di Marco, Valentina Panara, Gianna Sepede, Antonio Ferretti, Mauro Gianni Perrucci

**Affiliations:** ^1^Department of Neuroscience, Imaging and Clinical Sciences, “G. d'Annunzio” University of Chieti-Pescara, Chieti, Italy; ^2^Institute for Advanced Biomedical Technologies, “G. d'Annunzio” University of Chieti-Pescara, Chieti, Italy; ^3^Department of Medicine and Science of Aging, “G. d'Annunzio” University of Chieti-Pescara, Chieti, Italy; ^4^Section of Diagnostic Imaging and Therapy, Radiology Division, Department of Neuroscience and Imaging, “SS Annunziata” Hospital, “G. D'Annunzio” University, Chieti, Italy; ^5^Department of Basic Medical Sciences, Neurosciences and Sense Organs, University “A. Moro” Bari, Chieti, Italy; ^6^National Health Trust, Department of Mental Health, Chieti, Italy

**Keywords:** aging, brain plasticity, second language learning, cognitive decline, resting state, functional connectivity

In the original article, the statistically significant differences in the MMSE scores between the two groups were incorrect. A correction has been made to the **Results**, subsection **Cognitive Performances**, paragraph one:

Control and intervention subjects were evaluated at the baseline phase (T0) and at the end of the 4 months (T1) period for their neuropsychological abilities. Four subjects were excluded, one did not observe inclusion criteria [had periventricular nodular heterotopia (PNH)], two did not accept to be retested at post-training condition, and one did not attend a sufficient percentage of lessons. We observed slight differences between group in terms of age (Control group Mean: 65.7, SD 3.7; Intervention group: Mean 69.5, SD 5.3; One-Way ANOVA *F* = 4.42, *p* = 0.046) and education (Controls: Mean 13.0, SD 3.0; Intervention group Mean 9.6 SD 2.9; One-Way ANOVA *F* = 8.43, *p* = 0.0008). A detailed description of statistical analysis results can be seen in Table 1. The normality of the distribution was controlled by Kolmogorov–Smirnov test (Ksd *d* = 0.11, *p* > 0.20). Statistically significant differences in MMSE score were found within and between the two groups at both T0 and T1 (*p* = 0.009). In more details, the two groups significantly differ at T0, with the control group performing better than the intervention group (29.35 versus 27.23, Duncan *post hoc p* = 0.001); on the contrary, the between group difference disappeared at T1 (28.28 versus 27.81, Duncan *post hoc p* = 0.42). In fact, only the control group significantly decreased its performances over time (29.35 versus 28.28 Duncan *post hoc p* = 0.017), whereas the intervention group remained stable (27.23 versus 27.81) (see Table 3 and Figure 2).

The authors apologize for this error and state that this does not change the scientific conclusions of the article in any way. The original article has been updated.

