# Supplementary figures and images for: Effects of Second Language Learning on the Plastic Aging Brain: Functional Connectivity, Cognitive Decline, and Reorganization
Source: Front Neurosci. 2019 May 15;13:423. doi: 10.3389/fnins.2019.00423 (PMC6529595; doi:10.3389/fnins.2019.00423)

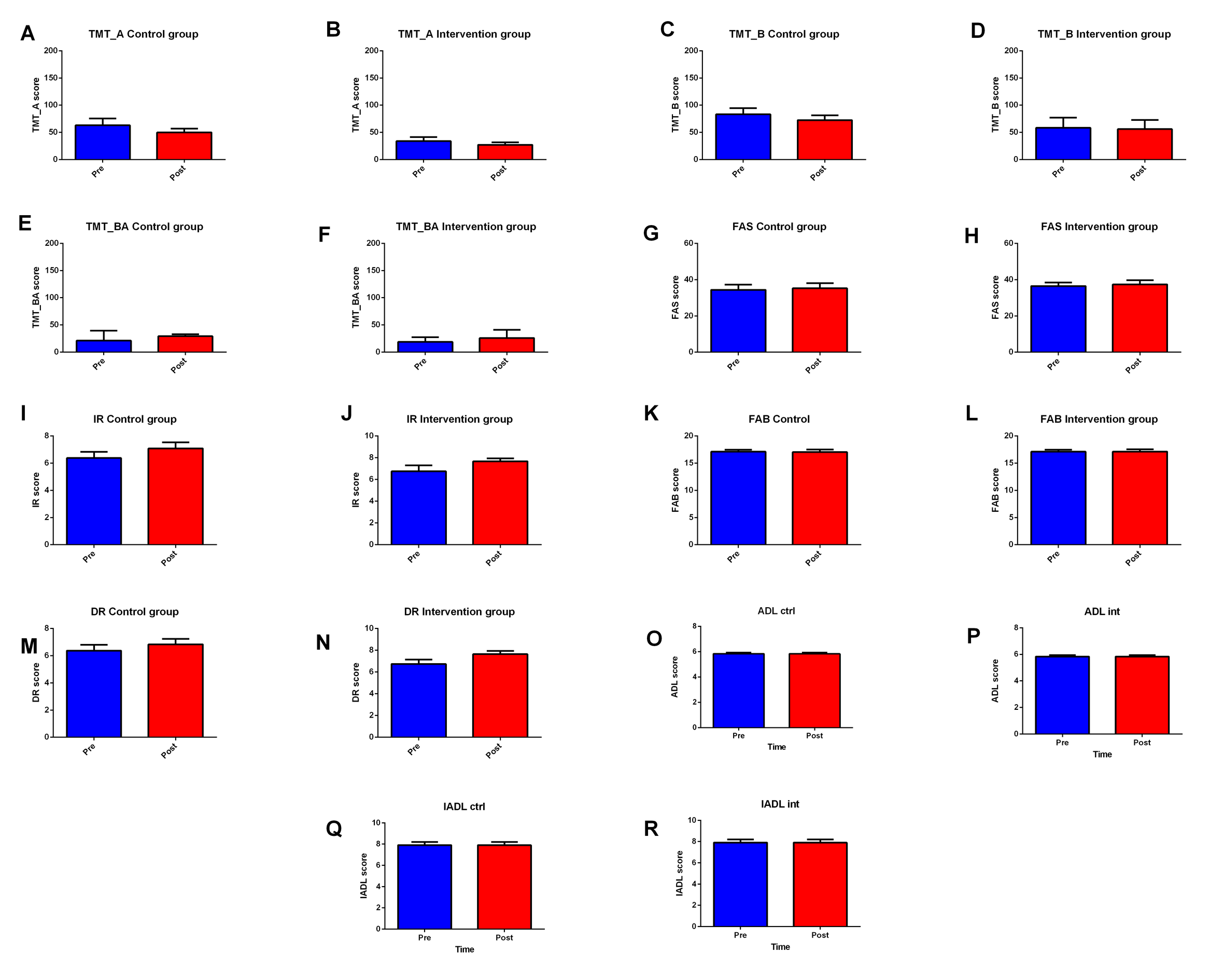

Supplement: FIGURE S1 — Second language learning affects cognitive performances. Histograms depict measures of neuropsychological evaluation in control and intervention groups at the beginning of the study (Pre) and after 4 months (Post). Graphs show results, expressed as means and SEM for the performance of both groups in their sustained attention skills using TMT_A test for controls (A) and interventions (B), divided attention (TMT_B) for controls (C), and interventions (D); task coordination and set-shifting (TMT_BA) for controls (E) and interventions (F). Global prose memory test (Babcock story, version A) was also performed, with immediate recall (IR) (I,J) and delayed recall (DR) subtest (M,N). Scores for functioning of frontal lobes (FAB) are represented for controls (K) and intervention group (L). Differences for scores for lexical production (FAS) are also displayed (G,H), together with ADL (O,P) and IADL scales (Q,R). [file Image_1.TIF]
